# Chemical lysis of cyanobacteria

**DOI:** 10.1186/s13036-015-0007-y

**Published:** 2015-06-05

**Authors:** Kunal K. Mehta, Niklaus H. Evitt, James R. Swartz

**Affiliations:** Department of Bioengineering, Stanford University, Stanford, CA 94305 USA; Department of Chemical Engineering, Stanford University, Stanford, CA 94305 USA

## Abstract

We have developed a mixture of enzymes and chemicals that completely lyse cyanobacteria. Since the treatment involves only readily-available chemicals and simple proteins that degrade the components of the cyanobacterial cell wall, it can easily be used in high-throughput applications requiring lysis for subsequent intracellular measurements. Our lysis technique consistently enables complete lysis of several different cyanobacterial strains, and we demonstrated that DNA, mRNA, and proteins are preserved in the lysates. Chemical lysis can be superior to existing techniques because of its convenience, reliability, and amenability to a variety of downstream applications.

**Electronic supplementary material:**

The online version of this article (doi:10.1186/s13036-015-0007-y) contains supplementary material, which is available to authorized users.

## Background

Significant efforts have been directed towards the fundamental goal of using photosynthesis to sustainably produce chemicals and fuels from sunlight and water, usually with either cyanobacteria or algae as hosts [1]. As the simplest photosynthetic organisms, cyanobacteria have attracted particular attention [2] for a range of applications including harvesting their remarkably wide library of natural products [3–5] and metabolic engineering for chemical production [6–10]. However, these advances have been limited by lower productivities in cyanobacteria than in traditional hosts. Two recent efforts to engineer cyanobacteria to produce chemicals by photosynthesis achieved productivities of 137 mg/L/day for isobutryaldehyde [11] and 391 mg/L/day for sucrose [12]. To our knowledge, these are the best productivities achieved from cyanobacteria in lab-scale cultures; however, they are almost two orders of magnitude lower than, for example, 14 g/L/day for succinate in *E. coli* [13] and 3.6 g/L/day for artemisinic acid in *S. cerivisiae* [14].

We believe high-throughput genetic engineering of cyanobacteria will be essential to overcome these limitations and enable the use of photosynthesis to sustainably synthesize chemicals. Applications for high-throughput genetic approaches include screening large libraries of parts [15] to optimize pathways and genetic mapping via insertional mutagenesis [16] to help determine genetic influences on observed phenotypes.

Cell lysis to enable intracellular measurements is a fundamental requirement in the strain engineering cycle. It is particularly challenging in cyanobacteria compared to traditional hosts. A recent assessment of several existing methods for cyanobacterial lysis demonstrated effective lysis only by using low-throughput methods such as sonication, mechanical disruption, or lyophilization [17]. A controllable lysis system has also been developed wherein a lytic casette from a bacteriophage, including a lysozyme and accessory proteins, was expressed in *Synechocystis* sp. PCC 6803 under the control of a nickel-inducible promoter [18], but this requires prior genetic engineering and is not generally adaptable. Thus, new and convenient techniques amenable to high-throughput analysis are needed.

**Fig. 1:**
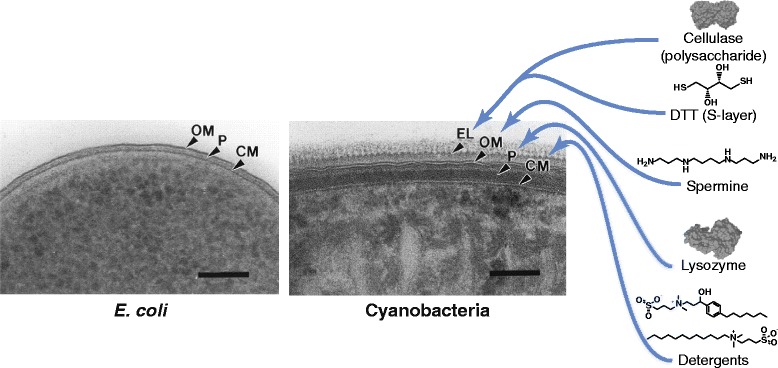
Micrograph of the cell wall of *E. coli* compared to that of a cyanobacterium, *Phormidium uncinatum*. The cyanobacterium has a much thicker peptidoglycan layer and a unique external layer containing an *S*-layer and exopolysaccharide. We tested a series of enzymes and chemicals meant to degrade each of these layers as shown at right. EL = external layer (*S*-layer and exopolysaccharide); OM = outer membrane; P = peptidoglycan; CM = cytoplasmic membrane. The detergents used were 3-(4-Heptyl)phenyl-3-hydroxypropyl)dimethylammoniopropanesulfonate (upper structure) and 3-(*N*,*N*-Dimethylmyristylammonio)propanesulfonate (lower structure). Scale bars are 100 nm. PDB IDs: 3LZM (lysozyme), 1OLQ (cellulase). The cell wall images were adapted from [20]

The major obstacle to lysis is the resiliance of the cell wall, which is thicker and more complex in cyanobacteria than in *E. coli* and many other commonly engineered organisms. The cyanobacterial cell wall contains a thicker and more highly crosslinked peptidoglycan layer [19], and cyanobacteria unlike *E. coli* have a surface layer (*S*-layer) composed of polymerized proteins and an exopolysaccharide coating [20] (Fig. 1). The *S*-layer of *Synechocystis*, one of the most widely-used model cyanobacteria, has only recently been characterized in detail [21].

We sought to develop a simple, one-step chemical process that would degrade each of these barriers systematically. Some reagents for degrading cell wall components are well-established, such as lysozyme for peptidoglycan and detergents to permeabilize membranes. However, since other components of the cyanobacterial cell wall have not been well characterized, we tested a variety of other reagents to ensure all the layers of the cell wall could be breached. Here, we describe the cloning and testing of a novel lysozyme from a cyanobacteria-specific bacteriophage and the development of a chemical treatment to fully lyse cyanobacteria in a high-throughput manner.

## Results

We first tested a lysozyme from a cyanobacteria-specific phage [22] for its ability to more effectively degrade the cyanobacterial peptidoglycan layer relative to currently used enzymes. Our hypothesis was that a lysozyme from cyanobacterial phage might be uniquely suited to degrading the cyanobacterial peptidoglycan layer and might even be capable of breaking through other barriers as well. A novel lysozyme gene (GenBank accession number: KR259643), selected from a family of 23 identified by homology matching of ORFs in DNA from a cyanobacteria-phage community collected in Yellowstone National Park [22], was cloned and purified in *E. coli*.

We initially sought to optimize reaction conditions for cyanobacterial lysis. In these experiments, we compared a chemical lysis process using cyanophage lysozyme to a mechanical lysis procedure using vortexed glass beads as the best established procedure for cyanobacterial lysis. We tested a range of temperatures (4–60 °C), reaction pH values (5.5–9.2), and reaction times (20–180 min). In the initial tests, to achieve any lysis, we had to complement the lysozymes with BugBuster, a commercial lysis reagent which we have previously used with *E. coli*. Lysis was quantified by measuring the absorbance at 660 nm of chlorophyll released from the cells during the reaction; the “extent of lysis” is plotted in terms of raw absorbance units measured in the supernatant after centrifugation.

A treatment with lysozyme and BugBuster at 30–42 °C and pH 6–8 for 90 min achieved the best results, although it did not enable complete lysis (see Additional file 1). In these initial experiments, we estimated that the maximum extent of lysis corresponded to approximately 50 % of the cells present, although the variability was significant.

**Fig. 2:**
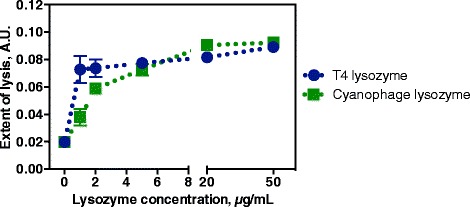
Comparison of cyanophage lysozyme with T4 lysozyme for cyanobacterial lysis. The concentration of cyanophage lysozyme (purified in-house) and T4 lysozyme (MCLab) was titrated to compare their effectiveness in lysing *Synechocystis* sp. PCC 6803. Each reaction contained lysozyme at the indicated concentration, 25 mM MES buffer (pH 6.2), and 1 X BugBuster reagent. Reactions were incubated for 90 min at 37 °C. We estimated that the maximum lysis achieved here corresponded to 50 % of total lysis; approx. 25 % of the cells were lysed with BugBuster alone. Error bars are standard deviations of triplicate reactions

A primary question was whether the new cyanophage lysozyme would perform better than other lysozymes against cyanobacteria. We tested this by comparing cyanophage lysozyme activity on *Synechocystis* sp. PCC 6803 to that of T4 lysozyme, a commercially available enzyme commonly used for *E. coli* lysis (Fig. 2). Overall, we found that cyanophage lysozyme was equivalent to T4 lysozyme in promoting cyanobacterial lysis (although at low lysozyme concentrations there was a slight advantage to using T4 lysozyme). Based on this, most subsequent experiments were done with T4 lysozyme.

Our initial tests revealed that lysozyme had to be supplemented with BugBuster to enable lysis. Since BugBuster is a proprietary mixture of unknown reagents, we sought to discover commercially available components that complement T4 lysozyme in breaching the outer barriers of cyanobacteria. Moreover, we found that BugBuster did not enable complete lysis under the optimized conditions mentioned above.

**Fig. 3:**
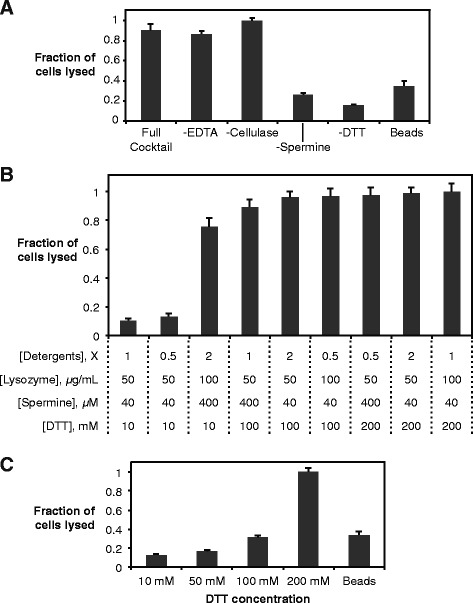
A chemical cocktail for cyanobacterial lysis. All reactions were shaken for 90 min at 37 °C. **a** Testing the contribution of EDTA, cellulase, spermine, and DTT to cell lysis. All samples contained T4 lysozyme at 10 μg/mL and the detergents C7BzO (0.1 % w/v) and SB3-14 (1 % w/v). The “full cocktail” also contained EDTA at 1 mM, *T. viride* cellulase at 50 μg/mL, spermine at 40 μM, and DTT at 200 mM; other samples omitted one of the reagents as indicated. Error bars are standard deviations of three independent experiments. **b** A design-of-experiments optimization of the levels of lysozyme, detergent, spermine, and DTT. The lysozyme was cyanophage lysozyme in this experiment. “1 X detergents” refers to 1 % C7BzO and 0.1 % SB3-14 (w/v). Error bars are standard deviations of triplicate reactions. **c** Influence of DTT concentration with other components as described for the “full cocktail” in (A), omitting EDTA and cellulase. Error bars are standard deviations of three independent experiments

In addition to lysozyme, we tested cellulases (to break down exopolysaccharide), EDTA (to destabilize the outer membranes), DTT (to break potential disulfide bonds in the cell wall), spermine (which was previously shown to improve the activity of T4 phage lysozyme [23]), and the detergents 3-(4-Heptyl)phenyl-3-hydroxypropyl)dimethylammoniopropanesulfonate (C7Bz0) and 3-(*N*,*N*-Dimethyltetradecylammonio)propanesulfonate (SB3-14) to permeabilize membranes. These detergents were previously found to be particularly beneficial for cell lysis [24]. Further rationale for these components and their concentrations is given in Additional file 1. Figure 3a shows data from a series of reactions in which one of these components was omitted to determine which are beneficial for cyanobacterial lysis. In these and subsequent experiments, we achieved complete lysis, judged by the lack of any residual pigment/color in the debris pellet after incubation and centrifugation of the cell suspension (see Methods). Thus, these data are normalized to the signal from the reactions that gave complete lysis and are plotted in terms of “fraction of cells lysed”.

Removing EDTA or cellulase had a minimal effect on lysis efficiency, but spermine and DTT were both important, with less than 50 % lysis activity without either. EDTA was originally included to help destabilize the outer membrane by chelating divalent cations that stabilize negatively-charged sugars; its lack of effect in both *Synechocystis* (Fig. 3a) and *E. coli* (Additional file 1) suggests that the detergents by themselves enable sufficient permeabilization of the membranes.

Removing cellulase, which was included to degrade the secreted exopolysaccharide layer, also had a minimal effect on lysis efficiency. This suggests that exopolysaccharide is not a significant barrier to lysozyme and the other additives. However, these experiments were performed on cells in exponential phase, and we suspected that exopoysaccharide would be thicker in stationary phase. Thus we also tested the effect of cellulase on late-stage cultures. Even in this case, cellulase did not increase the extent of lysis (Additional file 1), suggesting the EPS in general does not pose a significant barrier to other components. Based on these results, subsequent experiments were performed with T4 lysozyme, detergents, spermine, and DTT.

We next performed a design-of-experiments (DOE) analysis to optimize the concentrations of these four reagents (Fig. 3b) and potentially discover interactions between cyanophage lysozyme, detergents, spermine, and DTT. It was interesting that several of the combinations tested gave virtually complete lysis. We thought this might suggest an interaction between some of the components in the mixture, but the DOE analysis did not reveal any significant interactions. Marginal means analysis of the DOE results predicted that the optimal combination is 50 μg/mL cyanophage lysozyme, 2 X detergents, 400 μM spermine, and 200 mM DTT; subsequent experiments were done with this cocktail, except that 10 μg/mL T4 lysozyme was used instead of 50 μg/mL cyanophage lysozyme based on a separate optimization for that omponent (see Additional file 1).

Since the requirement for DTT at such a high concentration was particularly interesting, we titrated it separately while keeping the concentrations of the other components constant (Fig. 3c). This confirmed the results of the design-of-experiments, with a significant increase in lysis activity even from 100 to 200 mM. We examine the implications further in the Discussion.

**Fig. 4:**
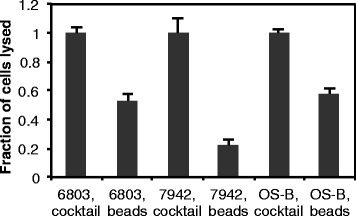
Lysis of *Synechocystis* sp. PCC 6803, *Synechococcus* sp. PCC 7942, and *Synechococcus JA-2-3B’a* (“OS-B”). The “cocktail” contained T4 lysozyme at 10 μg/mL, the detergents C7Bz0 (0.2 % w/v) and SB3-14 (2 % w/v), spermine at 400 μM, and DTT at 200 mM; “beads” refers to vortexing with glass beads (see Methods). Error bars are standard deviations of triplicate samples

To show the utility of our procedure with additional strains of cyanobacteria, we tested lysis of *Synechococcus* sp. PCC 7942, another widely-used model strain, and *Synechococcus* JA-2-3B′a, a strain isolated from a Yellowstone hot spring [22] (Fig. 4). Full lysis was obtained with each strain using the cocktail. Furthermore, comparison with the commonly used bead lysis procedure indicated that our new chemical procedure can provide two- to three-fold improvement in lysis and more reliably enables full lysis of these three strains, highlighting the observation that mechanical disruption can be an unreliable method for cyanobacterial lysis.

**Fig. 5:**
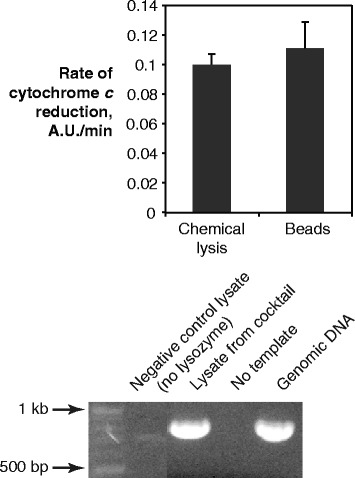
Protein activity and PCR amplification from chemical lysates. (*top*) Activity of ferredoxin-NADP^+^ reductase, as measured by reduction of ferredoxin and subsequent reduction of cytochrome *c*, was comparable in lysates produced from beads and our chemical cocktail. (*bottom*) 1 % agarose gel showing PCR amplification from genomic DNA of a 700 bp DNA fragment from lysates produced with our cocktail

We demonstrated the biological utility of lysates produced with our chemical cocktail in several ways. It is important that a lysis procedure preserve the activity of enzymes in the lysate. We chose ferredoxin-NADP^+^ reductase as an example enzyme, and demonstrated that its activity is preserved in our lysate using a cytochrome *c* reduction assay [25] (Fig. 5, top); the activity was comparable to that from a lysate produced by bead disruption. We also amplified an 800 bp genomic fragment via PCR, showing that genomic DNA was not degraded in our lysates (Fig. 5, bottom). Finally, we isolated mRNA (Quick-RNA miniprep kit, Zymo) and reverse-transcribed cDNA (Verso reverse-transcription kit, Thermo-Fisher) with comparable yields starting from lysates produced with our cocktail or with beads (not shown). These results suggest that our chemical cocktail preserves biological components in cyanobacterial lysates for a variety of applications.

## Discussion

Our chemical treatment uses detergents to permeabilize membranes in the cyanobacterial cell wall, spermine and DTT to weaken the external layer, and lysozyme to break down peptidoglycan. Initially, we thought a lysozyme from a cyanobacteria-specific phage might be uniquely suited to lysing cyanobacteria, but this was not the case.

Lysozymes cleave the linkage between *N*-acetylmuramic acid and *N*-acetylglucosamine in peptidoglycan, which is consistent across almost all bacteria. This could explain why T4 lysozyme worked as well as cyanophage lysozyme against cyanobacteria: Although the cyanobacterial peptidoglycan is thicker and more highly cross-linked than in other species, the actual target of the lysozyme is the sugar chain sequence, which is similar. Nevertheless, the overall cyanobacterial cell wall clearly presents a unique challenge, and a systematic approach was required to breach it.

DTT was the most surprising additive required for full lysis (Fig. 3c). It is generally used as a reducing agent. We initially thought the target might be disulfide bonds in the S-layer, but the *Synechocystis* S-layer protein has no cysteine residues [21]. Certain cell wall components in yeast are disulfide bonded to each other [26]; it is possible that similar disulfide bonds exist between other components of the *Synechocystis* cell wall – for example, between membrane proteins, particularly in the outer membrane. T4 lysozyme does not contain disulfide bonds [27]; combined with the fact that DTT does not significantly promote *E. coli* lysis, we do not believe DTT acts on the lysozyme. Moreover, the concentration of DTT that was required – at least 50 mM, with up to 200 mM for the full benefit – is far higher than that typically needed to reduce disulfide bonds. We note that the results of the DTT titration (Fig. 3c) indicating a requirement for 200 mM DTT seem to disagree with some of the individual conditions in the design-of-experiments (Fig. 3b). However, the requirement for 200 mM DTT was consistently reproducible over several independent experiments.

We considered the possibility that the requirement for a high concentration of DTT implies a physical effect, perhaps an intercalation in one of the layers of the cell wall, rather than a chemical effect. We tested this by substituting DTT (redox potential of −330 mV at pH 7) with β-Mercaptoethanol (redox potential of −260 mV) and TCEP (a stronger reducing agent than DTT). β-Mercaptoethanol was essentially as effective as DTT. TCEP also effectively contributed to cyanobacterial lysis, but the lysates had a red color instead of the yellow-green color normally seen; this decreased the absorbance measured at 660 nm and suggests that TCEP may reduce other molecules in the lysate, perhaps the pigments (data in Additional file 1). The fact that other reducing agents could replace DTT suggests that the function of DTT in the cocktail is indeed a reducing one (as opposed to a physical effect); further work is needed to determine why such a high concentration is required.

Spermine was also required for complete cyanobacterial lysis. Previous work with T4 lysozyme against *E. coli* [23] suggested that spermine directly enhances the muramidase activity of T4 lysozyme at spermine concentrations below 100 μM, and stabilizes spheroplasts at higher concentrations, counteracting that enhancement. However, we achieved virtually complete lysis of *E. coli* with lysozyme and detergent only, with small additional benefits from either EDTA or DTT. Since spermine is required for lysis of cyanobacteria but not *E. coli* using our cocktail, we believe its benefit here is through an interaction with a unique feature of the cyanobacterial cell wall and not with lysozyme. At our reaction pH, spermine is fully protonated and carries four positive charges, so it probably does not pass through either cell membrane; most likely, it acts on the exopolysaccharide or S-layer.

To verify biological activity in lysates produced by our cocktail, we tested enzyme activity (using ferredoxin-NADPH reductase as an example enzyme), DNA amplification by PCR, and isolation and reverse transcription of mRNA. We quantified lysis by measuring the release of pigments into the lysate; this necessarily means that the pigments were not damaged by the lysis cocktail. For proteins in particular, adsorption onto the large surface area presented by glass beads can be a problem, so a chemical procedure could provide a significant advantage for protein analysis. We note a potential limitation of our procedure in that the high concentration of DTT could break disulfide bonds in proteins.

At the scales we have used (reaction volumes ~ 100 μL; ~1 μg cells), we found that chemical lysis was significantly more effective than mechanical disruption with beads. We believe this is because mechanical disruption, especially for cells with such a strong cell wall, requires tremendous force often only provided by specialized equipment such as a BeadBeater homogenizer.

## Conclusions

We examined a fundamental operation in the strain engineering cycle, cell lysis, from the perspective of the unique cyanobacterial cell wall. As a result, we developed a chemical treatment that fully lyses several strains of cyanobacteria at physiologically relevant conditions and does so more effectively than mechanical disruption with glass beads.

Our optimized cocktail consists of T4 lysozyme at 10 μg/mL, the detergents C7Bz0 at 0.2 % w/v and SB3-14 at 2 % w/v, spermine at 400 μM, and DTT at 200 mM. This reliably enabled full lysis of three different cyanobacterial strains.

Our lysis procedure uses only chemical components, making it easily adaptible to high-throughput applications, and preserves genomic DNA, mRNA, and enzyme activity for further analysis. We believe this will expand the scope of intracellular measurements that are feasible in cyanobacteria.

## Methods

### Strains and plasmids

DNA fragments were joined by Gibson assembly [28] and propagated in *E. coli* strain DH5α, and proteins were expressed in *E. coli* strain BL-21 (DE3). Plasmids for *in vivo* expression use a pET21b backbone (Novagen), and plasmids for expression by cell-free protein synthesis use a pY71 backbone [29] modified with an XbaI restriction site between P_T7_ and the ribosome-binding site to allow for modification of the region coding for the 5′ end of the mRNA. In all constructs, the sequence of this region, starting 25 bp upstream of the RBS and extending through the first 15 bp of the coding sequence, was optimized for the mRNA to have a folding energy ΔG_f_ > −1.5 kcal/mol (using a custom MATLAB script, optimizeForCFPS.m, available at https://gist.github.com/kkmehta/0a1dbe272d7ff2aed011#file-optimizeforcfps-m) to minimize inhibition of translation initiation by 5′ secondary structure. Table 1 contains a list of plasmids constructed for this work. The gene sequence for cyanophage lysozyme 1 has been deposited in GenBank with accession number KR259643.

**Table 1:** Plasmids constructed for this work

Plasmid	Backbone::insert
pKM011	pET21b::cpL1
pKM012	pY71X::cpL1
pKM013	pET21b::cpL2
pKM014	pY71X::cpL2
pKM015	pET21b::cpL3
pKM016	pY71X::cpL3
pKM024	pET21b::strepII-GSA-cpL1
pKM025	pET21b::cpL1-GSA-strepII

The lysozyme gene used in this study was “cyanophage lysozyme 1” (cpL1; sequence provided in Additional file 1). Two other genes, cpL2 and cpL3, were also cloned, but did not express solubly in *E. coli*. The genes were cloned into either the pET21b vector for in vivo expression or the pY71X vector for cell-free expression (data not shown)

### Protein expression and purification

Proteins were expressed *in vivo* from BL21 (DE3) strains using IPTG induction. Briefly, 1 L cultures in terrific broth (Invitrogen) supplemented with ampicillin at 100 μg/mL were grown at 37 °C to OD ~1.5 and induced with IPTG at 100 μM. At this point, the temperature was reduced to 25 °C and proteins were expressed overnight. Cultures were harvested by centrifugation at 3000 × g and cells were lysed in a single pass through a high-pressure homogenizer (Avestin) at >18,000 PSI. Debris and insoluble material were removed by centrifugation at 23,000 × g. The soluble supernatant was purified on a streptactin column (IBA Life Sciences) according to the manufacturer’s protocol. After purification, elution fractions were pooled, concentrated ~30 X using a centrifugal filter device (Amicon Ultra, 10 kDa cutoff, Millipore), formulated in 20 % sucrose, flash frozen in liquid nitrogen, and stored at − 80 °C. Typical yields were 1 mg of purified protein from 3 (wet) grams of cells.

### Cyanobacterial lysis reaction

Unless otherwise stated, lysis reactions were carried out on *Synechocystis* sp. PCC 6803 (ATCC 27184) cells grown in BG-11 medium (Sigma) under constant illumination from fluorescent lamps at ~200 μE/m^2^/s while sparging a mixture of 5 % CO_2_ in air. Cells were harvested in exponential phase (culture OD_750_ of 0.5–1; doubling time 10–15 h). For lysis experiments, cells were pelleted by centrifugation and resuspended to OD 1.5 in MES buffer at pH 6.2 with lysozyme and either the specified lysis components (see Results) or 1 X BugBuster protein extraction reagent (Novagen). Reactions were conducted in either microcentrifuge tubes (300 μL reactions, shaken on a vortexer (vortex genie, VWR)) or deep-well 96-well plates (100–200 μL reactions, shaken on a Thermo Scientific titer plate shaker (shake setting 7)). Wells in the plate were sealed with a custom PDMS gasket to prevent cross-contamination while shaking. After the reaction, insoluble debris were removed by centrifugation at 10,000 × g for 5 min, and the degree of lysis was quantified by measuring absorbance of the supernatant at 660 nm, corresponding to a chlorophyll absorbance peak. Total lysis was confirmed by the absence of green color in the debris pellets, indicating that all cells had lysed and released their chlorophyll into the supernatant. Mechanical lysis via glass beads (150–200 μM in diameter; Sigma-Aldrich) was used as a comparison in many experiments, as this is the current standard for cyanobacterial lysis. In these reactions, beads at 25 % of the reaction volume [30, 31] were added to the cell suspension in buffer and detergents (to solubilize chlorophyll); these reactions were incubated and otherwise processed as described above.

All chemicals were obtained from Sigma-Aldrich. T4 lysozyme was obtained from MClab. Cellulases from *Trichoderma viride*, *Trichoderma reesei*, and *Aspergillus niger* (Sigma) were a kind donation from Lynette Cegelski. Design-of-experiments calculations were done in SPSS (IBM). The orthogonal design function was used to generate the set of experimental conditions, and a univariate general linear model was used to analyze the results.

## Additional file

Additional file 1:
**Coding sequence of cyanophage lysozyme, rationale for cocktail components and concenetrations, and results of additional optimization experiments.**
