# Supplementary material for: Chemical lysis of cyanobacteria
Source: J Biol Eng. 2015 Jun 5;9:10. doi: 10.1186/s13036-015-0007-y (PMC4478636; doi:10.1186/s13036-015-0007-y)
Supplement: Additional file 1: — Coding sequence of cyanophage lysozyme, rationale for cocktail components and concenetrations, and results of additional optimization experiments. [file 13036_2015_7_MOESM1_ESM.pdf]

Supplementary information to "Chemical Lysis of Cyanobacteria"

Kunal Mehta, Niklaus Evitt, James Swartz

Cyanophage lysozyme coding sequence

```
ATGCAGAGTATCGACTTCGGTCGTGCGCTGGACTTCACCCTGCGTTGGGAAGGCGGTTACGTTAACCACCCGT
CTGACCCTGGCGGTGCTACGAACCGTGGTATCACCCAGGTTACCTACAACCAGTGGCGTACCCAGAAAGGTCT
GCCGACGCGTGAAGTTCGTCTGATCGAAGAAGACGAGGTTCGCTCTATCTACTGGCAGTTCTATTGGGCGCCA
GTTGAAGGTCGTACCGCGCCTTCTTGGGTTCAGTTCCGTGTTTGCCTGTTCGACACCTTCGTACAGTTCGGTG
TTTTCGGTGGTACCTTCCTGTGGCAGAAAGTTTGCGGTGTTCCGGCGGACGGTCAGTGGGGTCCGGTTACTTC
TCGTGCCACCGAGAACCTGGTTTCTACCAAAGGTCCGCTGTGGTCTGGTATGGCGCTGGTTGGTGAACGTGTT
CGTTACCGTGCGCAGCGTGTTTCTCAGAACCGTTCTCAGCTGGCGTTCCTCCAGGGCTGGCTGAATCGTGATT
CTGATCTGCTGCTGTACCTGCTGAACCTGCGT
```

Cyanophage lysozyme optimized 5' UTR

```
ACGGAGGGAAATAAGATAACAAG AGGAGGT GAGAGT [ATG...]
```

CLUSTAL O(1.2.0) multiple sequence alignment of cpL1 with T4 lysozyme

```
cpL1 -----MQSIDFGRALDFTLRWEGGYVNHP---SDPGGATNRGITQVTYNQWRTQKGLPTR 52
T4   MNIFEMLRIDEGLRLKIYKDTEGYYTIGIGHLLTKSPSLNAAKSE-------LDKAIGRN 53
          *  ** *  *.:    ** *.         . : * . ::        :*.:  .

cpL1 EVRLIEEDEVRSIYWQFYWAPVEGR-----TAPS---WVQFRVCLF-DTFVQFGVFGG-- 101
T4   CNGVITKDEAEKLFNQDVDAAVRGILRNAKLKPVYDSLDAVRRCALINMVFQMGETGVAG 113
        :* :**...:: *   * *.*        *       .* * : : ..*:*  *

cpL1 ---------TFLWQKVCGVPADGQWGPVTSR-ATENLVSTKGPLWSGMALVGERVRYRAQ 151
T4   FTNSLRMLQQKRWDEAAVNLAKSRWYNQTPNRAKRVITTFRTGTWDAYKNL--------- 164
                 *::..   *..:*   * . *.. :.: :   *..   :

cpL1 RVSQNRSQLAFLQGWLNRDSDLLLYLLNLR 181
T4   ------------------------------ 164
```

29 identical residues (*)
20 strongly similar residues (:)
23 weakly similar residues (.)

Rationale for cocktail components/concentrations: We used EDTA to destabilize membranes by chelating divalent cations that normally stabilize negative charges in the phospholipid head groups; the concentration of 1 mM was chosen based on previous experience. DTT was used as a result of a previous experiment to determine whether cyanophage lysozyme has disulfide bonds (not shown here). It does not, but in the course of that work we found that it significantly promoted cyanobacterial lysis; those experiments tested a wide range of concentrations and based on those results a starting concentration of 100 mM was chosen for the current work. The "1 X" concentration of detergents was taken from the original patent describing them [1]. The initial concentration of 40 μM for spermine was taken from Jensen and Kleppe [2]. The concentration of cellulases was chosen based on the work of Yin et al. [3] and Zheng et al. [4].

As mentioned in the main text, the cyanophage lysozyme was a member of a family of 27 lysozyme genes discovered by Heidelberg et al. {Heidelberg:2009hb}. Three genes from the family were cloned and tested for soluble expression in *E. coli* (KKM PhD thesis, Stanford University, 2015), and the one we tested here gave the best soluble yield after expression and purification.

Optimization of pH and temperature for cyanobacterial lysis

All reactions contained 1 X BugBuster reagent and cyanophage lysozyme at 50 μg/mL (an excess, to ensure that peptidoglycan degradation was not limiting) and were incubated for 90 min. (*top*) pH optimization. The buffer was MES for pH 5.5 and 6.2, Tris for pH 7.3 and 8, HEPES for pH 8.5, and glycine for pH 9.2. (*bottom*) Temperature optimization. Above 42 °C, background levels of pigment release increased significantly.

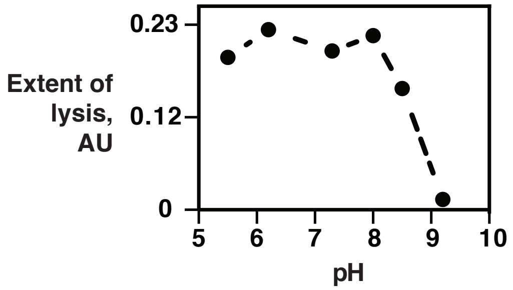

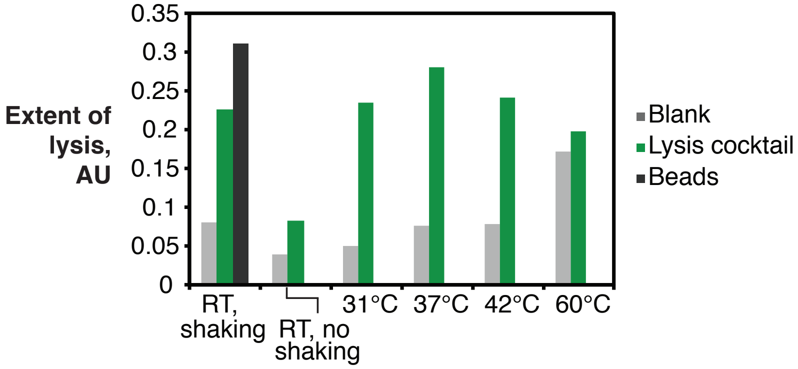

Example of pellets where no lysis has occurred (left) and total lysis (right)

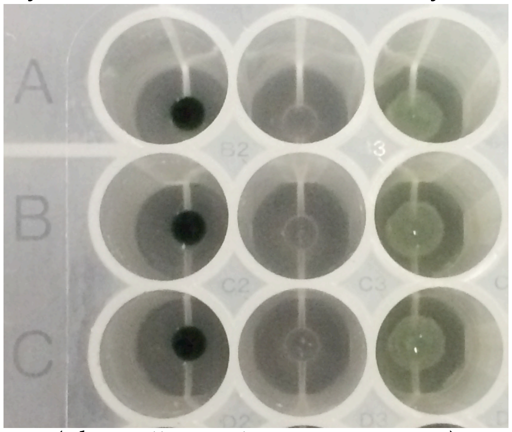

(The wells in column 2 are empty)

Time course of the lysis reaction at 37 °C:

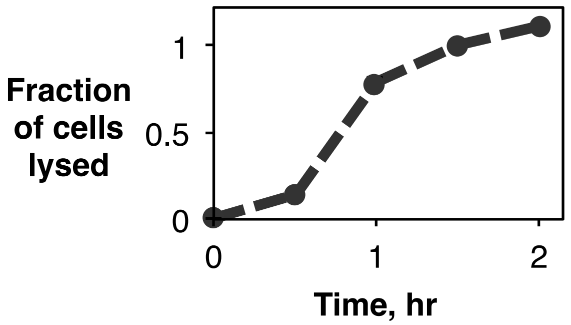

Time course at lower temperatures:

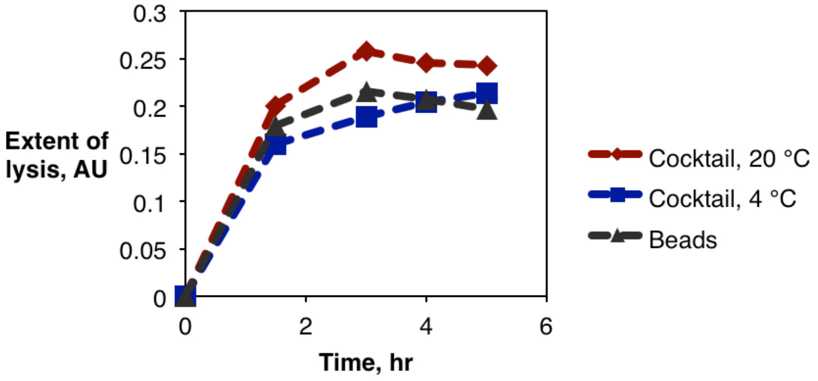

DTT and spermine have no effect on *E. coli* lysis:

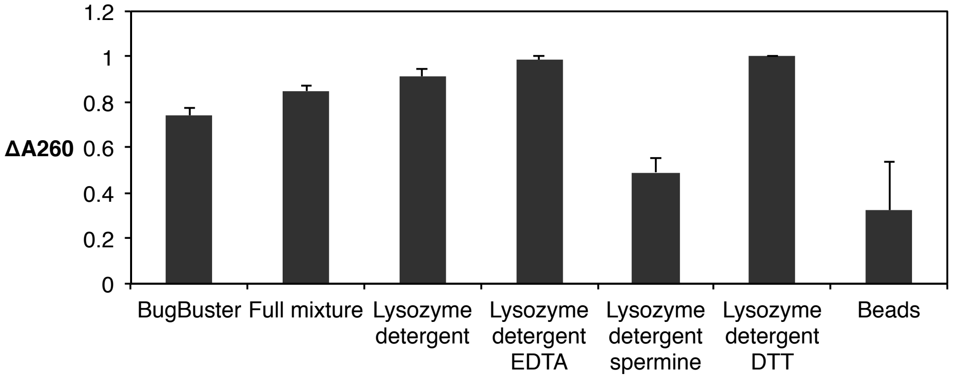

Concentrations: BugBuster, 1X; Lysozyme, 10 μg/mL, Sigma detergents mix, 2X; EDTA, 1 mM; spermine, 400 μM; DTT, 200 mM. The "full mixture" is lysozyme, detergent, EDTA< spermine, and DTT. Lysis was quantified by measuring absorbance of DNA released into the supernatant at 260 nm. Error bars are standard deviations of three independent experiments.

Cellulase does not help in exponential or stationary phase:

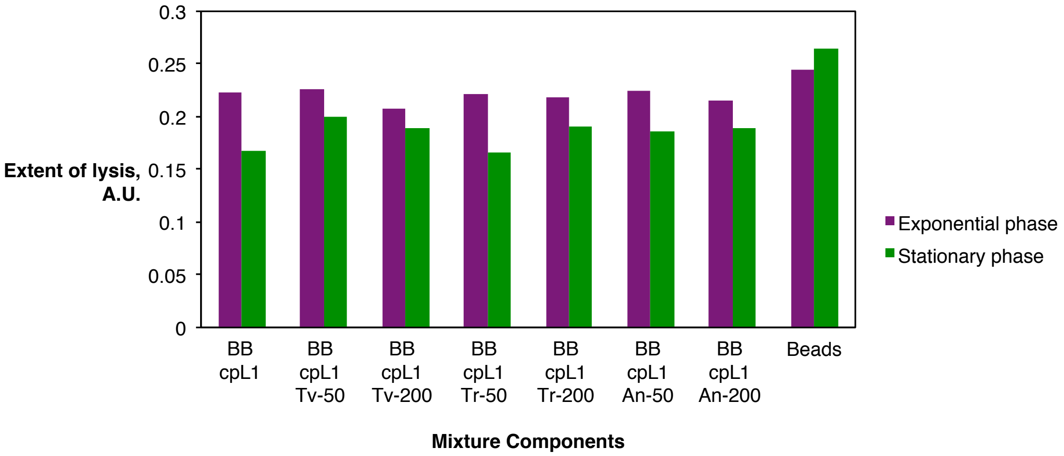

Abbreviations: "BB", 1 X BugBuster mix; "cpL1", 50 μg/mL cyanophage lysozyme; Tv, *T. viride* cellulase, 50 or 200 μg/mL; "Tr", *T. reesei* cellulase; "An", *A. niger* celluase. The beads sample did not achieve full lysis; green color was visible in the pellet.

Titration of T4 lysozyme using the optimized cocktail:

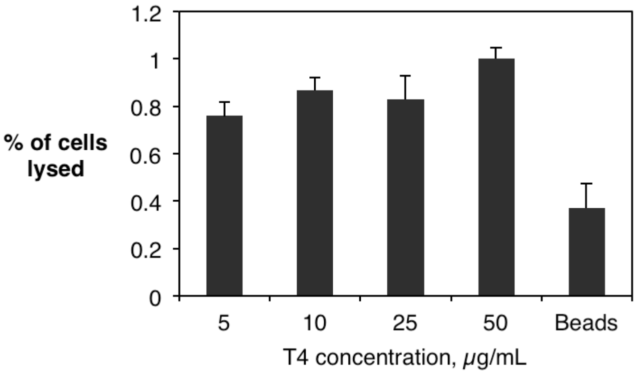

Error bars are standard deviations of triplicate reactions.

Replacing DTT with β-mercaptoethanol or TCEP as the reducing agent:

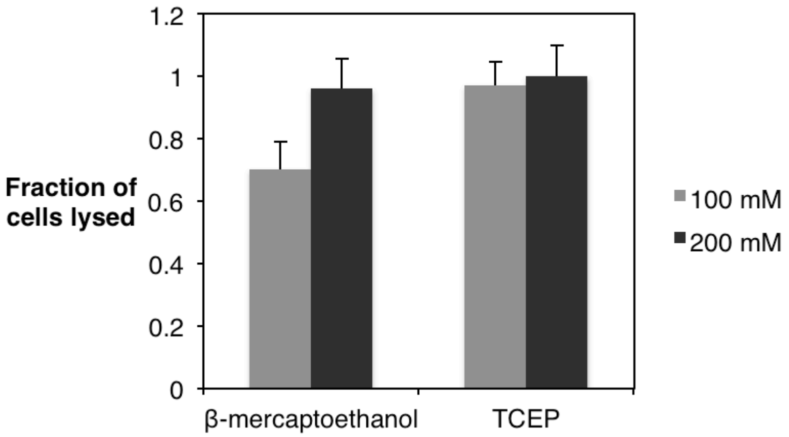

References

1. Porter J, Mehigh R: **Method for Extracting a Target Product from a Host Cell Employing Zwitterionic Detergent Combinations**. 2011.

2. Jensen HB, Kleppe K: **Effect of ionic strength, pH, amines and divalent cations on the lytic activity of T4 lysozyme.** *Eur J Biochem* 1972, **28**:116–122.

3. Yin L-J, Jiang S-T, Pon S-H, Lin H-H: **Hydrolysis of Chlorella by Cellulomonas sp. YJ5 cellulases and its biofunctional properties.** *J Food Sci* 2010, **75**:H317–23.

4. Zheng H, Yin J, Gao Z, Huang H, Ji X, Dou C: **Disruption of Chlorella vulgaris cells for the release of biodiesel-producing lipids: a comparison of grinding, ultrasonication, bead milling, enzymatic lysis, and microwaves.** *Appl Biochem Biotechnol* 2011, **164**:1215–1224.
